# Efficacy of simple and very brief handgrip and isometric exercises for reducing withdrawal symptoms in cigarette smokers: A pilot randomized controlled trial

**DOI:** 10.18332/tid/187839

**Published:** 2024-05-24

**Authors:** Min Jin Zhang, Yee Tak Derek Cheung, Qi Wang, Christopher Chi Wai Cheng, Tzu Tsun Luk, Wan Jia He, Man Ping Wang, Tai Hing Lam

**Affiliations:** 1 School of Nursing, the University of Hong Kong, Hong Kong, China; 2 School of Graduate Studies, Lingnan University, Hong Kong, China; 3 School of Public Health, the University of Hong Kong, Hong Kong, China

**Keywords:** exercise, handgrip, tobacco craving, very brief intervention, withdrawal symptom

## Abstract

**INTRODUCTION:**

Withdrawal symptoms lead to smoking relapse and reduce the intention to quit. The present pilot RCT examined the effect of simple and very brief handgrip and isometric exercises on reducing withdrawal symptoms, measured by the strength of tobacco craving, Questionnaire of Smoking Urges-Brief (QSU-B), Mood and Physical Symptoms Scale (MPSS), and Positive and Negative Affect Schedule (PANAS).

**METHODS:**

In this 2-arm, open-labeled pilot RCT, 30 current smokers who had abstained from tobacco for at least 9 hours were randomly assigned (allocation ratio 1:1) to either the intervention group that watched a 5-minute video and did 5-minute handgrip and isometric exercises (pulling and pushing) or control group that watched 10-minute healthy-diet videos. Measurements were taken before, immediately after, and 10 minutes post-intervention. Outcomes were self-reported strength of tobacco craving, QSU-B, MPSS, and PANAS scores. The effect size for group-by-time interaction was assessed using Cohen’s f^2^ (small=0.02, medium=0.15, large=0.35).

**RESULTS:**

Group-by-time interactions showed that the intervention group showed larger reductions than the control group in the strength of tobacco craving (Cohen’s f^2^=0.54, 95% CI: 0.52–0.57), QSU-B (Cohen’s f^2^=0.77; 95% CI: 0.74–0.80), and MPSS (Cohen’s f^2^=0.51; 95% CI: 0.46–0.56) over the three measurement points.

**CONCLUSIONS:**

This RCT showed that simple and brief handgrip and isometric exercises could immediately reduce withdrawal symptoms and up to 10 minutes.

**CLINICAL TRIAL REGISTRATION:**

in https://clinicaltrials.gov/

**IDENTIFIER:**

NCT04059497

## INTRODUCTION

Smoking is a major modifiable risk factor of early mortality, contributing to approximately 7.7 million deaths globally in 2019^1^. The risk of developing smoking-related health complications, such as heart disease and lung cancer, decreases as the duration of cessation increases^1^. Nonetheless, quitting is difficult as smokers often experience withdrawal symptoms when they start to quit. Withdrawal symptoms due to a deficit of nicotine usually include tobacco craving, stress, anxiety, tension, poor concentration, irritability, negative affect, and restlessness^2,3^. These symptoms increase the likelihood of smoking relapse and reduce quitting intention^4^. Exercise is a non-pharmacological intervention for smoking cessation, given its capacity to alleviate withdrawal symptoms and cravings. A recent systematic review has indicated that aerobic exercises exceeding 10 minutes reduce the desire to smoke, and moderate to high-intensity aerobic exercises significantly reduce the strength of this desire^5^. Additionally, short-bout (5–10 min) isometric exercise, which involves maintaining a static muscle contraction without moving the affected joint, is also effective in relieving withdrawal symptoms^6,7^, suggesting a spectrum of exercise durations and intensities is beneficial in smoking cessation.

Moderate- to high-intensity exercise for reducing nicotine withdrawal symptoms can be explained by the affect, biological, and cognitive mechanisms^8,9^. According to the affect hypothesis, smokers with temporary abstinence experience an increase in emotional stress, depression, anxiety, and anger^10^. Exercise helps regulate mood, reduce anxiety^11^, and acutely decreases sympathetic response to emotional stress, affective distress, and anxiety during the abstinence period^12^. The biological hypothesis posits that exercise activates the caudate nucleus, parietal lobe, parahippocampal gyrus, and fusiform gyrus, associated with reward processing and visuospatial attention, thereby reducing attentional bias toward smoking images^13^. For the cognitive mechanism, exercise enhances self-control^14^, perceived coping ability^15^, and self-esteem^16^, which indirectly increases smokers’ capacity to quit smoking.

Isometric exercise involves repetitive muscle contraction, short-term body pain, and instant heat, which might suppress smoking urges. The reduction in smoking urge is potentially mediated by mechanisms such as distraction and stress reduction^9^. A previous RCT showed that a 10-minute isometric exercise involving various static muscular contractions (i.e. pushing) and mindfulness could immediately reduce the desire to smoke and poor concentration for up to 5 and 15 minutes, respectively^6^. Handgrip, an affordable (as low as HK$12; US$1=HK$7.8) and portable tool, offers a simple and easy-to-use method that encourages smokers to exercise as a temporary coping strategy for combating withdrawal symptoms. The simplicity of using the handgrip could facilitate adherence to the exercise. Gripping a handgrip statically without releasing it is an isometric exercise, while repeatedly and rapidly gripping and releasing a handgrip leads to quicker fatigue, soreness, and pain. This is due to repeated and rapid contractions increasing metabolic demand in the muscles, swiftly depleting energy stores, and accumulating lactate, a metabolic byproduct contributing to the sensation of fatigue^17^. Such fatigue, soreness, and pain may offer immediate relief to tobacco cravings through mechanisms of distraction and stress reduction. Handgrip exercise, if proven to be effective in reducing withdrawal symptoms, could be a useful quitting aid for smokers.

To date, we have found that only our previous pilot RCT tested the effect of handgrip and isometric exercises on tobacco abstinence at 6-month follow-up among smokers using smoking cessation services^18^. In the present pilot RCT, we adopted a short-bout ‘10-second exercise’ (10s-E) to help smokers relieve tobacco cravings efficiently, as tobacco cravings typically persist for several minutes^18^. The 10s-E includes repeatedly and rapidly gripping and releasing a handgrip at least 30 times in 10 seconds for each hand, along with hand pushing and pulling, each also for 10 seconds. Hand pushing and pulling, which have been found to reduce tobacco cravings^6,7,18^, offer alternative options when a handgrip is not available. Participants are instructed to continuously repeat the 10s-E for a total duration of 5 minutes. Our previous pilot RCT showed that reported doing handgrip and isometric exercises when craving was associated with more tobacco abstinence^18^. However, the intervention did not specify the required exercise amount or measure the participants’ exercise duration^18^. The mechanism and the immediate effect of 10s-E on reducing withdrawal symptoms and how long the exercise effect can be sustained remain unclear. Moreover, the previous pilot RCT focused on smokers enrolled in smoking cessation services, which limited the intervention’s generalizability. Many smokers attempt to quit on their own without professional support and may experience different challenges and levels of motivation.

The present pilot RCT aimed to assess the effect of an intervention of 10s-E on reducing withdrawal symptoms in adult smokers who had temporary abstinence. The hypothesis was that the exercise intervention is effective in reducing the strength of tobacco craving, desire and anticipation of smoking, mood, and physical symptoms, and increasing positive affect and reducing negative affect immediately and up to 10 minutes, compared with the control group.

## METHODS

### Study design

The present study was an open-labeled, two-arm pilot RCT (allocation ratio 1:1). Participants were randomly assigned to either: 1) the intervention group, where they watch a 5-minute exercise video and do 10s-E for 5 minutes; or 2) the control group, where they watched healthy-diet videos for 10 minutes. Measurements were taken at baseline, pre-intervention, immediately postintervention, and 10 minutes post-intervention. Ethical approval was granted by the Institutional Review Board of the University of Hong Kong/Hong Kong Authority Hong Kong West Cluster (IRB reference no: UW-19-950). We followed CONSORT for reporting the pilot RCT. The trial has been registered with ClinicalTrials. gov (Clinical trial registration: NCT04059497 in https://clinicaltrials.gov/).

### Participants

Eligibility was determined via a two-stage exhaled carbon monoxide (CO) validation process. In the first stage, eligibility criteria included: 1) consume at least ten conventional cigarettes a day, a criterion chosen to target smokers who are likely to experience intense withdrawal symptoms during temporary abstinence^19^; 2) aged ≥18 years; 3) able to communicate in Cantonese; 4) self-reported no serious injury of hands and arms; 5) no reported mental illnesses; and 6) exhaled CO ≥15 parts per million (ppm), a threshold based on previous research examining the acute effects of tobacco withdrawal to identify smokers with higher cigarette consumption, validated by Bedfont *piCO+*^TM^ Smokerlyzer^6,20^. The second stage required participants to abstain from tobacco products for at least 9 hours, given that the half-life of exhaled CO varies from 2 to 8 hours^21^. This was verified by a reduction in exhaled CO levels to <15 ppm and at least a 50% decrease from their initial measurements.

### Procedures

From September 2019 to January 2020, potential participants were identified and proactively recruited from three sources: 1) smoking hotspots, where smokers who gathered around ashtrays in outdoor public areas and smoked; 2) the Tung Wah Group of Hospital Integrated Center on Smoking Cessation; and 3) open recruitment through mass emails of the University of Hong Kong. All potential participants were invited to enroll for the initial eligibility screening.

Eligible individuals were invited to complete a self-administrated baseline questionnaire and join the face-to-face session located at smoking cessation clinics or our research office. At this scheduled face-to-face session, participants who passed the second exhaled CO validation were invited to sign the written consent form, complete a self-administered pre-intervention questionnaire, and be randomly allocated to the intervention or control group. The intervention group watched a 5-minute exercise video followed by doing 5 minutes of 10s-E. The control group watched 10 minutes of healthy-diet videos. Both groups completed three measurements (T1, pre-intervention; T2, immediately post-intervention; and T3, 10 minutes post-intervention), with the entire session taking about 40 minutes. After completing all interventions and assessments, all participants were given HK$350 (about US$45) to compensate for their time and travel expenses.

### Randomization

Simple individual randomization was done by serially numbered, opaque, and sealed envelopes (SNOSEs) with a card indicating the group (either intervention or control) inside. A researcher (YTDC, not involved in participant recruitment) generated a random sequence list of the group allocation with a random number generator and serially numbered identifiers for these envelopes. After obtaining consent, the research assistant opened a SNOSE following the number sequence to ensure allocation concealment. Blinding was not possible as all participants received behavioral intervention, but self-administered questionnaires measured outcomes without interference from the research assistant.

### Intervention

*Intervention group*

In the face-to-face session, each participant in the intervention group was provided with a handgrip tailored to their grip strength, ranging from 10 to 25 kg, and viewed the 5-minute exercise video (10s-E) demonstrated by a researcher (THL). The 10s-E exercise video demonstrates three main activities: 1) rapid grip and release of the handgrip about 30 times in 10 seconds in each hand; 2) hand pushing for 10 seconds; and 3) hand pulling for 10 seconds (Supplementary file Figure 1). After the video, a research assistant guided the participants to repeat the 10s-E five times in the next 5 minutes and told them they could slow down if they felt pain in their hands. Between each exercise set, 10–20 seconds of rest time was allowed.

*Healthy-diet control group*

Two videos on how to maintain a healthy diet (i.e. low intake of sugar and salt, more fruits and vegetables), which lasted for about 10 minutes (Supplementary file Figures 2 and 3), were shown to control group participants to control the non-specific effects of distraction or researchers’ attention, as well as equal time duration between intervention and control groups.

After completing the self-administrated post-intervention (T2) questionnaire, both groups were shown the same two health education videos for about 10 minutes on: 1) nicotine dependence and withdrawal symptoms; and 2) how to prepare healthy food at home (Supplementary file Figures 4 and 5); and completed the 10-minute post-intervention (T3) self-administered questionnaire. These videos aimed to distract the participants’ attention while waiting for the T3 assessment.

### Sample size calculation

The sample size calculation was based on the strength of tobacco craving, which was rated in 7 levels from ‘not at all’ to ‘extremely’. We conducted an *a priori* power analysis using G*Power Software for independent samples Student’s t-test^22^, assuming the group difference in mean change of the score is 2 (standard deviation 1.3)^7^ and aiming to detect a significance level of 1% and statistical power of 90%, a total sample size of 30 participants (15 in each group) was needed.

### Outcomes

Participants’ sociodemographic characteristics, daily cigarette consumption, quit attempts, future plan to quit, Fagerström test for nicotine dependence^23^, grip strength (assessed with a dynamometer), and physical activity level (International Physical Activity Questionnaires; IPAQ)^24^ were collected by the selfadministered baseline questionnaire.

Withdrawal symptoms included the strength of tobacco craving, desire and anticipation of smoking, mood, and physical symptoms, and positive and negative affect, which were assessed via self-administered questionnaires from T1 to T3. We chose to measure the outcomes at 10 minutes as the final follow-up measurement because a previous similar study showed that the effect of similar exercises on reducing the desire to smoke diminished after about 5 minutes, and the effect on sustained concentration declined at about 15 minutes^6^.

The strength of tobacco craving was assessed with a single item: ‘How strong is your desire to smoke right now?’, which was answered in 7 levels from ‘not at all’ to ‘extremely’, with test-retest reliability (intraclass correlation coefficient, ICC=0.62; 95% CI: 0.42–0.78) in the present study.

However, a single item of the strength of tobacco craving might not reflect the underlying desire and motivation to use tobacco. Therefore, we used the 10-item Questionnaire of Smoking Urges-Brief (QSU-B) to measure the desire and anticipation of smoking^25^. This scale was validated in Chinese smokers and yielded a very good internal consistency (Cronbach’s alpha=0.96)^26^. This scale comprises two subscales: 5 items on desire (i.e. ‘a cigarette would taste good now’) and five items on anticipation (i.e. ‘smoking would make me less depressed’). Each question can be answered in 5 levels, from ‘strongly agree’ to ‘strongly disagree’. The Cronbach’s alpha of QSU-B ranged from 0.83 to 0.95 at the three time points in the present study.

Other withdrawal symptoms were measured by the Mood and Physical Symptoms Scale (MPSS), including seven items: ‘irritable’, ‘restless’, ‘hungry’, ‘poor concentration’, ‘stress’, ‘tension’, and ‘depressed’. The component ‘craving to smoke’ was removed as it overlapped with the strength of tobacco craving. Each question can be answered in 5 levels, from ‘strongly agree’ to ‘strongly disagree’. The MPSS has been widely used to assess transient shifts in mood and physical symptoms from lack of nicotine and has shown good psychometric properties^27^. Acceptable internal consistency was reported both by the scale authors^27^ (Cronbach’s alpha=0.78) and in the present study (Cronbach’s alpha ranged from 0.78 to 0.89 at the three time points).

Moods and emotions were measured by the 20-item Positive and Negative Affect Schedule (PANAS)^[Bibr cit0028]28^, including ten positive adjectives and ten negative adjectives. Each question can be answered in 5 levels from ‘strongly agree’ to ‘strongly disagree’. Positive and negative affect subscales range from 10 to 50. The PANAS was validated in the Chinese population and showed high internal consistency^29^. In the present study, Cronbach’s alpha ranged from 0.74 to 0.79 for the positive affect scale and 0.60 to 0.65 for the negative affect scale at the three time points.

At T2, we also assessed the intervention group participants’ perception of the coached exercise video, perceived knowledge, perceived efficacy, confidence to do 10s-E, plan to do 10s-E, perceived difficulty, and intention to recommend the exercise to others. For participants in the control group, we also assessed their perception of the first healthy-diet video, perceived knowledge, perceived efficacy, confidence to do the healthy diet, plan to do the healthy diet, perceived difficulty, and intention to recommend a healthy diet to others.

### Statistical analyses

The normality of baseline continuous variables was assessed using skewness-kurtosis tests. Baseline characteristics between the intervention and control groups were compared using Fisher’s exact test for binary and categorical variables, independent sample t-tests for normally distributed continuous variables, and Mann-Whitney U tests for non-normally distributed continuous variables; p-values from these tests were for reference only as all differences were due to chance.

The effect sizes of within-group pre-post changes were assessed with Hedges’ g (bias-corrected Cohen d) and its 95% CI. Hedges’ g of 0.2, 0.5, and 0.8 indicates a small, medium, and large effect size, respectively. Linear mixed models (random intercept only), including group, time, and group-by-time interaction, were used to examine the outcome change from T1 to T2 and T3 between intervention and control groups. The original scales for all dependent variables were applied in the linear mixed models because estimates for fixed effects obtained from linear mixed models remain relatively unbiased even when the distribution of dependent variables deviates from normality^30^. The residual variance of the null model with only the intercept and random effects and the residual variance of the model that included both fixed and random effects were used to calculate the proportion of variance of the outcome explained by the predictors in the full model. These data were further used to calculate Cohen’s f^2^ based on the formula of Selya et al.^31^. Cohen’s f^2^ of 0.02, 0.15, and 0.35 indicates a small, medium, and large effect size, respectively^32^. All analyses were done using STATA (Version 15). A two-sided p<0.05 indicated statistical significance.

## RESULTS

From September 2019 to January 2020, of the 123 potential participants screened for eligibility, 76 were eligible at intervention sessions and 30 provided consent for participation. Two were recruited from smoking hotspots, 2 recruited from the Tung Wah Group of Hospital Integrated Center on Smoking Cessation, and 26 participants recruited by mass e-mail. No participant dropped out, and thus there were no missing data (Supplementary file Figure 6). Table 1 shows that about 80% of the participants were male and the mean age was 39.1 years (SD=13.4). Participants in both groups reported high levels of physical activity. All baseline characteristics were similar between the intervention and control groups.

**Table 1:** Baseline characteristics of the intervention and control groups (N=30)

*Characteristics*	*Intervention group (N=15) % (n)*	*Control group (N=15) % (n)*	*p*
**Age** (years), mean (SD)	39.9 (3)	38.3 (4)	0.75^a^
**Sex** (male)	13 (87)	12 (80)	0.50
**Education level Bachelor’s or higher**	9 (60)	7 (47)	0.36
**Marital status**			0.36
Married	9 (60)	7 (47)	
Single/divorced	6 (40)	8 (53)	
**Not living with children**			0.30
Yes	14 (93)	12 (80)	
No	1 (7)	3 (20)	
**Employment Hired/self-employed**	12 (80)	9 (64)	0.30
**Monthly household income** ≥HK$30000 (US$1=HK$7.8)	9 (60)	10 (67)	0.40
**Type of housing** (private housing)	9 (60)	11 (73)	0.35
**Daily traditional cigarette consumption,** median (IQR)	15 (6)	14 (5)	0.31^b^
**Abstinence hours before the intervention,** median (IQR)	13 (5)	12 (3)	0.46^b^
**Exhaled CO at enrolment** (ppm), mean (SD)	29.0 (11.6)	26.2 (12.7)	0.53^a^
**Exhaled CO before intervention** (ppm), mean (SD)	9.2 (2.7)	9.5 (4.8)	0.85^a^
**Ever had quit attempt**	10 (67)	13 (87)	0.20
**Plan to quit smoking**			0.46
Quit within 6 months	6 (40)	4 (27)	
Quit after 6 months	3 (20)	3 (20)	
Not decided yet	6 (40)	8 (53)	
**FTND score**, mean (SD)	4.4 (1.9)	3.6 (1.9)	0.25^a^
**Right hand grip strength** (kg), mean (SD)	40.7 (9.4)	38.8 (8.2)	0.57^a^
**Left hand grip strength** (kg), mean (SD)	36.3 (9.8)	35.3 (8.5)	0.77^a^
**IPAQ** High	9 (60)	10 (67)	0.50

FTND: Fagerström test for nicotine dependence. IPAQ: International Physical Activity Questionnaire. The smokers who scored high on the IPAQ were those who engaged in vigorous intensity activity on at least 3 days achieving a minimum total physical activity of at least 1500 MET minutes a week, or had ≥7 days of any combination of walking, moderate intensity or vigorous intensity activities achieving a minimum total physical activity of at least 3000 MET minutes a week (a MET is a multiple of the estimated resting energy expenditure).

a p for independent t-test.

b p from Mann-Whitney test. IQR: interquartile range.

**Figure 1:**
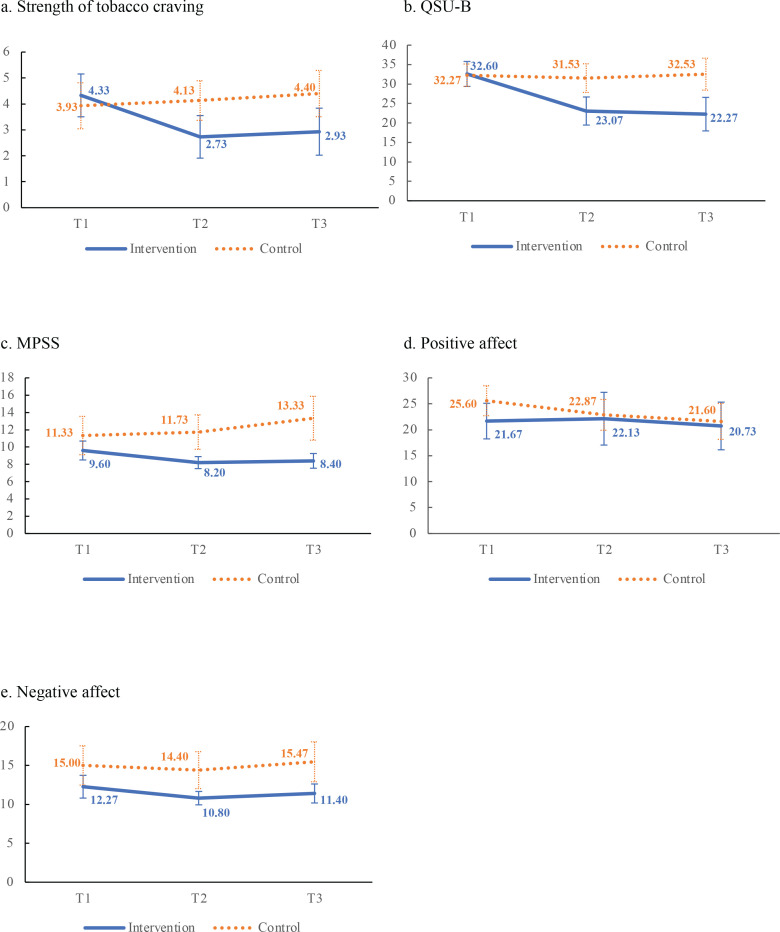
Mean score ± 1.96 standard error of all outcomes in the 3 measurement time points (N=30)

Table 2 shows that participants in both groups reported moderate the strength of tobacco craving, moderate desire and anticipation of smoking, low levels in mood and physical symptoms and negative affect, and moderate positive affect at T1. Figure 1 and Table 2 show that in the intervention group from T1 to T2, significant reductions in the strength of tobacco craving (within-group Hedges’ g effect size, ES= -0.99; 95% CI: -1.74 – -0.23), QSU-B (Hedges’ g ES= -1.41; 95% CI: -2.21 – -0.61), and MPSS (Hedges’ g ES= -0.77; 95% CI: -1.52 – -0.03). From T1 to T3, significant reductions in the strength of tobacco craving (Hedges’ g ES= -0.82; 95% CI: -1.56 – -0.07) and QSU-B (Hedges’ g ES= -1.38; 95% CI: -2.17 – -0.58) were observed. The control group showed no significant within-group differences in the strength of tobacco craving, QSU-B, MPSS and PANAS. Table 2 also shows significant group-by-time interactions and great effect sizes in reduction in the strength of tobacco craving (Cohen’s f^2^=0.54, 95% CI: 0.52–0.57), QSU-B (Cohen’s f^2^=0.77; 95% CI: 0.74–0.80), and MPSS (Cohen’s f^2^=0.51; 95% CI: 0.46–0.56), suggesting that the experimental group had greater reduction in the strength of tobacco craving, QSU-B, and MPSS over time, than the control group. However, there were no significant group-by-time interactions in PANAS.

**Table 2:** Within- and between-group differences of strength of tobacco craving, QSU-B, MPSS, positive and negative affect (N=30)

*Outcomes (score range)*	*Intervention group (N=15)*					*Control group (N=15)*					*Group × time interaction*
	*T1 Mean (SD)*	*T2 Mean (SD)*	*T1 vs T2 Hedges’ g ES (95% CI)*	*T3 Mean (SD)*	*T1 vs T3 Hedges’ g ES, (95% CI)*	*T1 Mean (SD)*	*T2 Mean (SD)*	*T1 vs T2 Hedges’ g ES (95% CI)*	*T3 Mean (SD)*	*T1 vs T3 Hedges’ g ES (95 % CI )*	*Cohen’s f ^2^ (95 % CI )*
**Strength of tobacco craving** (1–7)	4.33 (1.63)	2.73 (1.62)	**-0.99 (-1.74 – -0.23)**	2.93 (1.79)	**-0.82 (-1.56 – -0.07)**	3.93 (1.75)	4.13 (1.51)	0.12 (-0.59–0.84)	4.40 (1.76)	0.27 (-0.45–0.99)	**0.54 (0.52–0.57)**
**QSU-B** (10–50)^a^	32.60 (6.34)	23.07 (7.13)	**-1.41 (-2.21 – -0.61)**	22.27 (8.51)	**-1.38 (-2.17 – -0.58)**	32.27 (5.69)	31.53 (7.29)	-0.11 (-0.83–0.60)	32.53 (8.09)	0.04 (-0.68–0.75)	**0.77 (0.74–0.80)**
Desire (5–25)	17.73 (3.47)	12.60 (4.21)	**-1.33 (-2.12 – -0.54)**	12.87 (5.04)	**-1.12 (-1.89 – -0.35)**	17.33 (3.31)	17.87 (4.40)	0.14 (-0.58–0.86)	17.73 (4.40)	0.10 (-0.61–0.82)	**0.65 (0.63–0.67)**
Anticipation (5–25)	14.87 (3.44)	10.47 (3.36)	**-1.29 (-2.08 – -0.51)**	9.40 (3.79)	**-1.51 (-2.32 – -0.70)**	14.93 (2.79)	13.67 (4.03)	-0.36 (-1.09–0.36)	14.80 (4.30)	-0.04 (-0.75–0.68)	**0.78 (0.74–0.82)**
**MPSS** (7–35)^b^	9.60 (2.16)	8.20 (1.37)	**-0.77 (-1.52 – -0.03)**	8.40 (1.68)	-0.62 (-1.35 – 0.11)	11.33 (4.37)	11.73 (3.94)	0.10 (-0.62–0.81)	13.33 (5.01)	0.43 (-0.30–1.15)	**0.51 (0.46–0.56)**
Depressed (1–5)	1.40 (0.63)	1.13 (0.52)	-0.47 (-1.19–0.26)	1.13 (0.35)	-0.53 (-1.26–0.20)	1.53 (0.83)	1.47 (0.74)	-0.08 (-0.80–0.63)	1.67 (0.98)	0.15 (-0.56–0.87)	0.002 (-0.008–0.003)
Irritability (1–5)	1.33 (0.62)	1.07 (0.26)	-0.55 (-1.28–0.18)	1.13 (0.35)	-0.40 (-1.12–0.33)	1.53 (0.83)	1.33 (0.62)	-0.27 (-0.99–0.45)	1.47 (0.92)	-0.07 (-0.78–0.65)	0.003 (-0.002–0.005)
Restlessness (1–5)	1.53 (0.64)	1.20 (0.41)	-0.30 (-1.02–0.42)	1.33 (0.62)	-0.32 (-1.04–0.40)	1.67 (0.82)	1.73 (0.96)	0.07 (-0.65–0.78)	2.13 (1.06)	0.49 (-0.24–1.21)	0.09 (-0.09–0.10)
Hunger (1–5)	1.33 (0.82)	1.40 (0.63)	0.10 (-0.62–0.81)	1.33 (0.62)	0.00 (-0.72–0.72)	1.87 (1.19)	2.27 (1.34)	0.30 (-0.42–1.02)	2.40 (1.35)	0.42 (-0.31–1.14)	0.009 (-0.01–0.03)
Poor concentration (1–5)	1.40 (0.51)	1.40 (0.63)	0.00 (-0.72–0.72)	1.27 (0.46)	-0.27 (-0.99–0.45)	1.67 (0.90)	1.93 (0.88)	0.29 (-0.43–1.01)	2.27 (0.96)	0.65 (-0.09–1.38)	**0.11 (0.01–0.20)**
Tension (1–5)	1.07 (0.26)	1.00 (0.00)	-0.38 (-1.10–0.34)	1.07 (0.26)	0.00 (-0.72–0.72)	1.33 (0.62)	1.47 (0.64)	0.22 (-0.50–0.94)	1.60 (0.83)	0.37 (-0.35–1.09)	-0.02 (-0.01–0.03
Stress (1–5)	1.53 (0.92)	1.00 (0.00)	**-0.82 (-1.56 – -0.07)**	1.13 (0.35)	-0.58 (-1.31–0.16)	1.73 (0.96)	1.53 (0.74)	-0.23 (-0.95–0.49)	1.80 (0.78)	0.08 (-0.64–0.80)	0.03 (0.02–0.04)
Positive affect (10–50)^c^	21.67 (6.78)	22.13 (10.04)	0.05 (-0.66–0.77)	20.73 (9.07)	-0.12 (-0.83–0.60)	25.60 (5.71)	22.87 (5.87)	-0.47 (-1.20–0.25)	21.60 (6.84)	-0.64 (-1.37–0.10)	0.37 (-0.23–0.39)
Negative affect (10–50)^c^	12.27 (2.89)	10.80 (1.70)	-0.60 (-1.31–0.12)	11.40 (2.41)	-0.33 (-1.05–0.39)	15.00 (4.99)	14.40 (4.69)	-0.12 (-0.84–0.59)	15.47 (5.07)	0.09 (-0.62–0.81)	-0.01 (-0.04–0.02)

T1: before intervention. T2: immediately after intervention. T3: 10 minutes after intervention. ES: effect size. Effect sizes for within-group difference were summarized as Hedges’ g, and effect size for group × time interaction estimated as Cohen’s f^2^ obtained from linear mixed model. Hedges’ g of 0.2, 0.5, and 0.8 indicate a small, medium, and large effect size, respectively. Cohen’s f^2^ of 0.02, 0.15, and 0.35 indicate a small, medium, and large effect size, respectively.

a QSU-B: Questionnaire of Smoking Urges-Brief.

b MPSS: Mood and Physical Symptoms Scale.

c Positive and negative affect were measured by the 20-item Positive and Negative Affect Schedule (PANAS).

Table 2 shows in the intervention group, from T1 to T2, significant reductions in the subscales of QSU-B ‘desire’ (Hedges’g ES= -1.33; 95% CI: -2.12 – -0.54) and ‘anticipation’ (Hedges’g ES= -1.29; 95% CI: -2.08 – -0.51), and ‘stress’ of MPSS (Hedges’g ES= -0.82; 95% CI: -1.56 – -0.07). From T1 to T3, both QSU-B subscales ‘desire’ (Hedges’g ES= -1.12; 95% CI: -1.89 – -0.35) and ‘anticipation’ (Hedges’g ES= -1.51; 95% CI: -2.32 – -0.70) showed significant reduction. Table 2 also shows from T1 to T3, the score of ‘poor concentration’ in MPSS increased greatly in the control group, while the score did not change in the intervention group. The group-by-time interactions were significant with great effect sizes in subscale of QSU-B ‘desire’ (Cohen’s f^2^=0.65; 95% CI: 0.63–0.67) and ‘anticipation’ (Cohen’s f^2^=0.78; 95% CI: 0.74–0.82), and with small effect size in ‘poor concentration’ of MPSS (Cohen’s f^2^=0.11; 95% CI: 0.01–0.20).

Table 3 shows significantly higher scores in the intervention group than the control group in the question: ‘How satisfied are you with the exercise/healthy-diet video(s)?’ (p=0.041), and ‘How much do you understand the exercise/healthy-diet intervention?’ (p<0.001). Table 3 also shows no significant between-group differences in perceived efficacy of the video for smoking cessation, confidence to do the 10s-E/healthy diet for smoking cessation, plan to do the exercise/healthy diet, perceived difficulty of following the exercise/healthy diet for smoking cessation, and intention to recommend the video to others.

**Table 3:** Participants’ satisfaction towards the handgrip exercise and healthy diet intervention (N=30)

*Variables*	*Scale*	*Intervention group (N=15)*	*Control group (N=15)*	*p ^a^*
How satisfied are you with the exercise/healthy-diet video(s)?, median (IQR)	1–5^b^	4.0 (1.0)	3.0 (1.0)	**0.041**
How much do you understand the exercise/healthy-diet intervention?, mean (SD)	0–10^c^	8.0 (1.8)	3.7 (2.5)	**<0.001**
Would you agree that the exercise/healthy-diet intervention help quitting?, median (IQR)	1–5^d^	4.0 (1.0)	3.0 (2.5)	0.12
Confidence to follow the 10s-E or healthy-diet later on, median (IQR)	0–10^e^	6.5 (2.3)	5.0 (3.0)	0.34
Have a plan to do 10s-E or health diet, mean (SD)	0–10^f^	5.5 (2.2)	4.4 (2.3)	0.18
Perceived difficulty to follow the 10s-E or Healthy-diet to quit smoking, median (IQR)	0–10^g^	5.0 (2.0)	7.0 (4.0)	0.051
Intention to introduce the video to other people, n (%)		12 (80.0)	11 (73.3)	0.41

a p from the Mann Whitney U test and chi-squared test, comparing the scores and proportions of participants in the intervention and control group.

b 1=strongly dissatisfied to 5=strongly satisfied.

c 0=not understand at all to 10=totally understand.

d 0=disagree very much to 5=agree very much.

e 0=not at all confident to 10=extremely confident.

f 0=have no plan at all to 10=have a full plan. g 0=not at all difficult to 10=extremely difficult.

## DISCUSSION

We have demonstrated that watching a 5-minute 10s-E exercise video and doing 5 minutes of 5 rounds of simple and very brief 10s-E, significantly reduced the strength of tobacco craving, desire and anticipation of smoking in smokers who had temporary abstinence. The effect sustained at 10-minute follow-up with medium to large effect sizes. However, the 10s-E did not significantly increase positive affect and reduce negative affect, as the changes in these outcomes were negligible.

Our findings suggested two potential pathways of how the 10s-E can reduce withdrawal symptoms. Firstly, we are the first to show that our 10s-E intervention was effective in greatly reducing the strength of tobacco craving and maintaining concentration compared to the control group. Our findings were consistent with another RCT, which showed that 5 minutes of isometric exercises, including two circuits of fist clenching, pushing palms, squeezing thighs, and pressing the feet onto the floor, significantly reduced tobacco craving and maintained concentration for up to 5 and 15 minutes, respectively, compared to sitting passively^6^. Our control condition, including video watching for distraction purposes, suggested that the mechanism underlying the observed effect of handgrip and isometric exercise on maintaining concentration was probably not due to distraction, which is consistent with another RCT showing that the distraction effect was present in both exercise and non-exercise treatments^33^.

Second, our exercise intervention effectively reduced the desire and anticipation of smoking and hence reduced smokers’ positive expectancy of smoking. Similar effects were found in another RCT that aerobic exercise could significantly reduce desire and anticipation of smoking in smokers with temporary abstinence^34^. Therefore, handgrip, isometric exercise, and aerobic exercise potentially produce similar impacts to suppressing smoking desire and reducing smokers’ perceived rewards of smoking. The underlying mechanism may also be similar to the biological mechanism by which exercise can activate the caudate nucleus, parietal lobe, parahippocampal gyrus, and fusiform gyrus, which could reduce attentional bias toward smoking cues^13^, thereby reducing cravings and emotional attachment toward smoking-related stimuli.

Our present pilot RCT has a few clinical implications. Firstly, the 10s-E can quickly reduce tobacco craving and withdrawal symptoms, and smokers can experience the benefits immediately up to about 10 minutes after exercise. Nicotine replacement therapy (NRT) gum requires 30 minutes to show that effect^35^. Therefore, including 10s-E with or without NRT in smoking cessation promotion and services can potentially attract more smokers to try a new method and experience relief from tobacco craving and other withdrawal symptoms quickly. Second, compared to other exercises that require specific equipment and multiple supervised sessions, the 10s-E can be learned using one teaching video and a brief practical session. The video, as similar demonstration materials, can be easily shown in smoking cessation clinics, outreach campaigns, and social media for wide dissemination and downloaded to the smokers’ mobile phones for further use. Lastly, our findings have shown that 10s-E was well accepted and understood by the participants as a tool to assist quitting. In Hong Kong and elsewhere, smokers mostly prefer ‘self-determination’ than using intensive smoking cessation services as the method of quit attempt^36^. Our previous pilot study has shown that about 36% of smokers actively engaged in doing 10s-E when experiencing craving^18^. Therefore, this simple and very brief isometric exercise can be developed to facilitate unassisted quitting, that most smokers prefer, as many are not motivated to spend much time on exercises or use smoking cessation pharmacotherapy.

### Limitations

Our study has some limitations. First, non-blinding intervention and self-reported measurements might produce bias, which is unavoidable in such behavioral intervention. Second, our participants were not representative of all tobacco users as they were mainly cigarette-smoking males with 9-hour abstinence before the intervention, and more than half had a high level of physical activity. This limits the generalizability of our findings across all tobacco users. Therefore, further studies that include more diverse tobacco users are warranted. Third, our trial design of including a distraction component for the control group did not examine the independent effect of distraction on withdrawal symptoms, although previous studies^6,33^ had shown that the effect of exercise on reducing withdrawal symptoms was probably not due to distraction. Fourth, the follow-up period of only 10 minutes does not fully capture the enduring nature of withdrawal symptoms, considering that withdrawal symptoms may last for more than 10 minutes. This necessitates future studies with extended follow-up to assess the lasting effects of the intervention. Furthermore, the effect sizes of change in positive and negative affect due to the exercise were small, which might be due to the ceiling effect, as many participants reported no or mild affect problems at baseline. On the other hand, these null results could indicate that social desirability bias in the outcome measurements should be small.

## CONCLUSIONS

The simple and very brief handgrip and isometric exercises for 5 minutes, immediately and effectively reduced the strength of tobacco craving, desire and anticipation of smoking, and mood and physical symptoms in smokers who had temporary abstinence. The effects were sustained at 10 minutes. The approach of the 10s-E can be implemented in smoking cessation programs or practiced by smokers who want to quit smoking unassisted, as an easy and convenient quitting method. Smokers can use this method in daily life when they feel the craving for tobacco or to prevent these problems after stopping smoking for some time. Such a method can be easily promoted to attract more smokers to try quitting in smoking cessation clinics and outreach campaigns.

## Supplementary Material



## Data Availability

Data used in this study are owned by The University of Hong Kong and available upon request.
