# Supplementary material for: Efficacy of simple and very brief handgrip and isometric exercises for reducing withdrawal symptoms in cigarette smokers: A pilot randomized controlled trial
Source: Tob Induc Dis. 2024 May 24;22:10.18332/tid/187839. doi: 10.18332/tid/187839 (PMC11118784; doi:10.18332/tid/187839)
Supplement: Supplementary file 1 [file TID-22-87-s1.pdf]

Supplementary file Figure 1 Exercise video for the intervention group:

Video 1: 10-second exercise for combating smoking craving (4 minutes and 24 seconds)

Link: www.youtube.com/watch?v=mZex2Wwy3fU

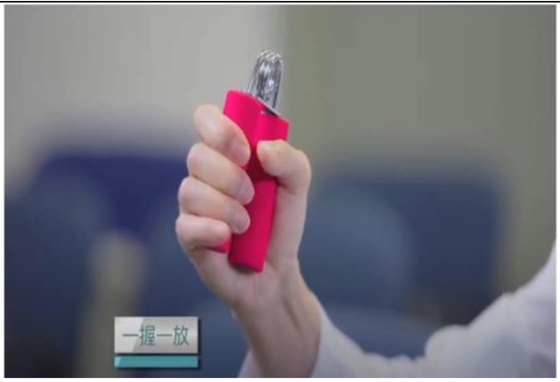<br><br>Push and pull the handgrip repetitively	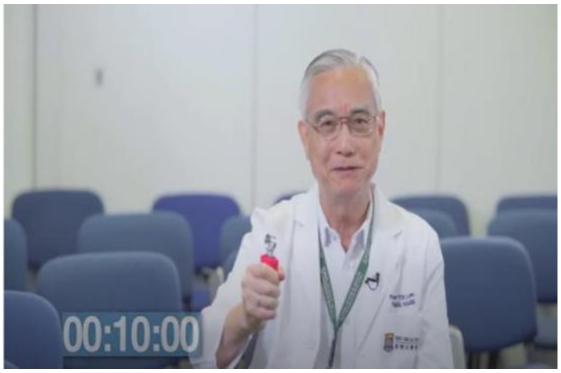<br><br>Practice the handgrip exercise for 10 seconds
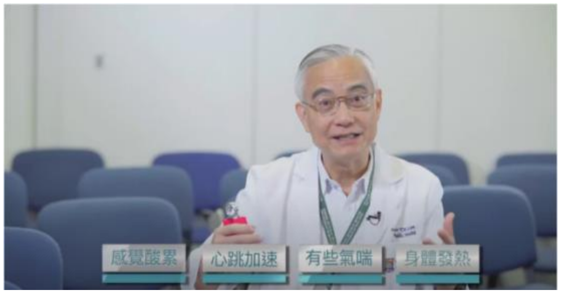<br><br>Practice until you feel a little tired, heart beat fast, breathe deeply to pant and warm	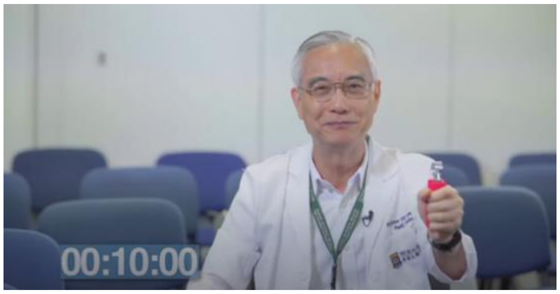<br><br>Switch hand to practice the handgrip for 10 seconds
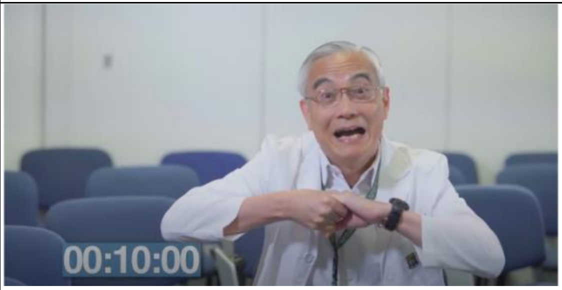<br><br>Pull hands for another 10 seconds	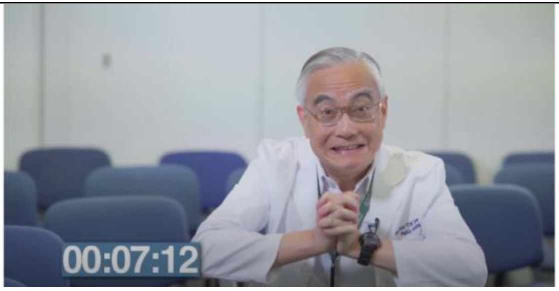<br><br>Push hands for another 10 seconds

Supplementary file Figure 2 Healthy diet video for the control group:

Video 1: Control the intake of sugar and salt for a healthy diet (5 minutes and 14 seconds)

Link: www.youtube.com/watch?v=3v1vF_zrpAc

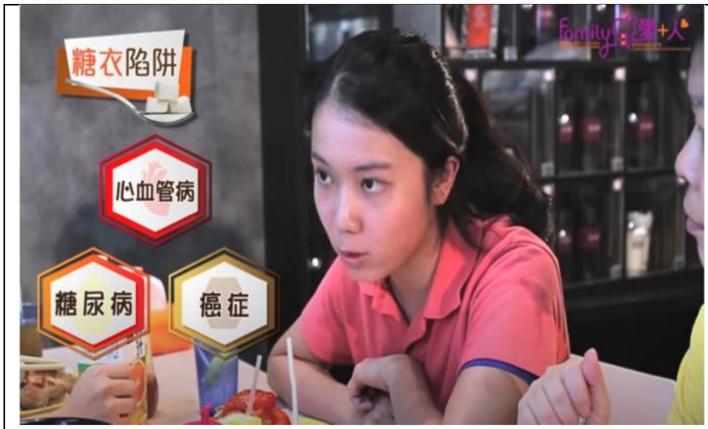

Consume too much sugar can lead to cardiovascular disease, diabetes, and cancer.

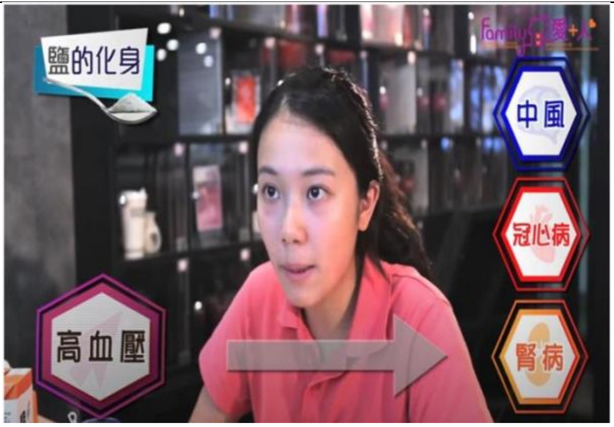

Salty food can lead to hypertension, stroke, coronary heart disease, and kidney disease

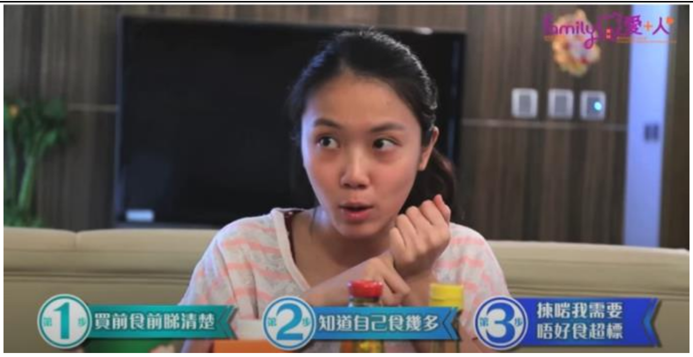

Healthy tips: 1) check the labels of the food before eating; 2) know how much we consume; and 3) only eat what we need, do not eat too much.

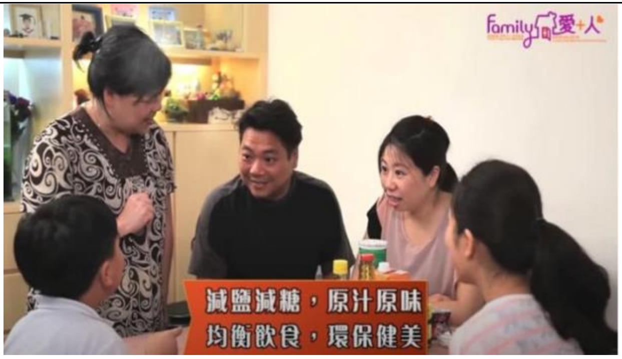

Eat less sugar and salt, eat more fruits and fresh vegetables to keep healthy.

Supplementary file Figure 3 Healthy diet video for the control group:

Video 2: Intake sugar in a healthy way (12 minutes and 19 seconds)

Link: https://www.youtube.com/watch?v=w_u4BYV2Okc).

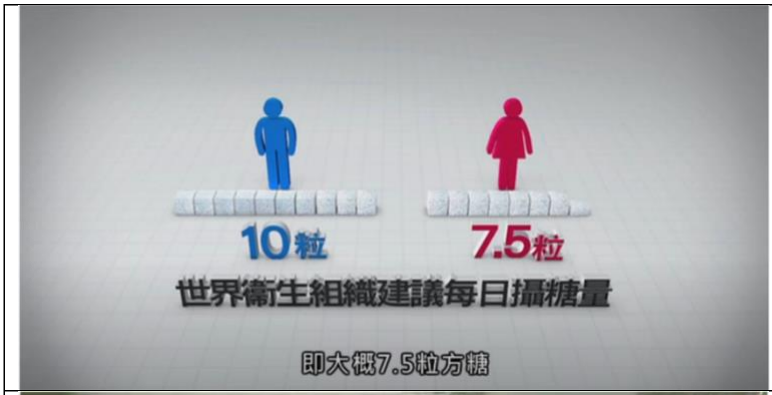	Amount of daily sugar intake suggested by the World Health Organization
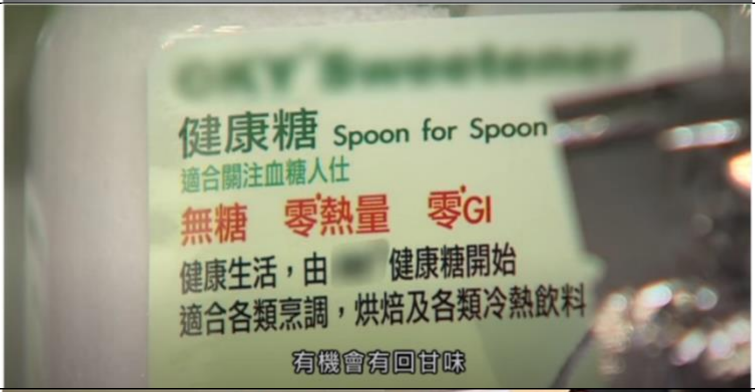	Use of healthy sugar for special population
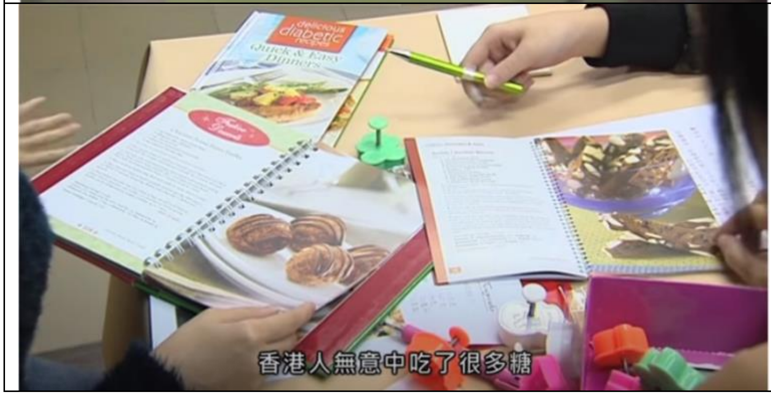	Self-made healthy desert for people with diabetes.

Supplementary file Figure 4 Health education video 1 for the intervention group and control group after completing the post-intervention questionnaire

Video 1: Nicotine addiction explained (1 minute 51 seconds)

Link: https://www.youtube.com/watch?v=NpbxHj3_qns)

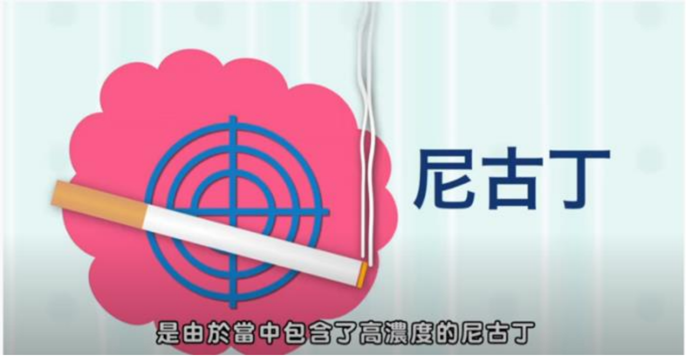

Introduction for how nicotine can be addictive

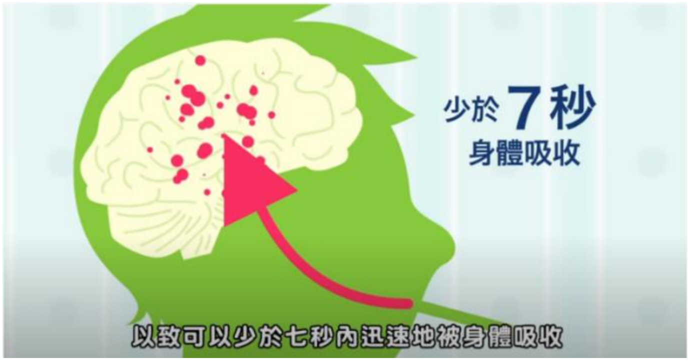

Absorption of nicotine

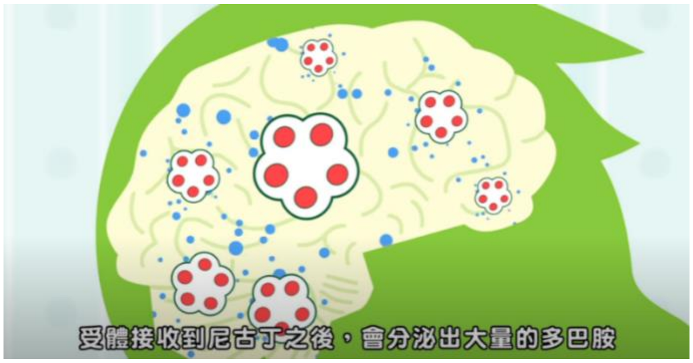

Function of nicotine in the brain

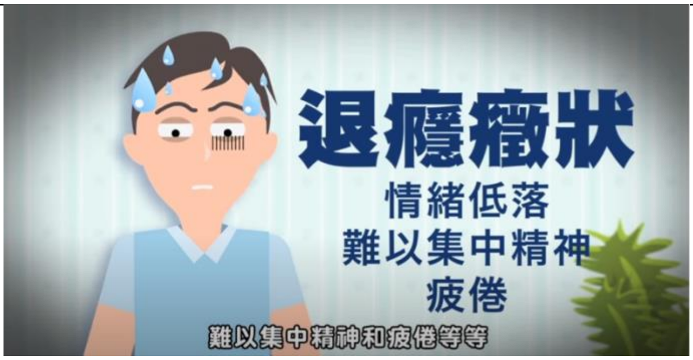

Introduction of withdrawal symptoms

Supplementary file Figure 5 Health education video 2 for the intervention group and control group after completing the post-intervention questionnaire

Video 2: Intake of healthy ingredients (7 minutes and 48 seconds)

Link: https://www.youtube.com/watch?v=V4wyRsl8mls

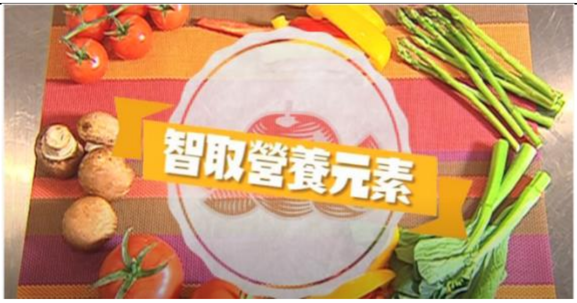

Intake of healthy food

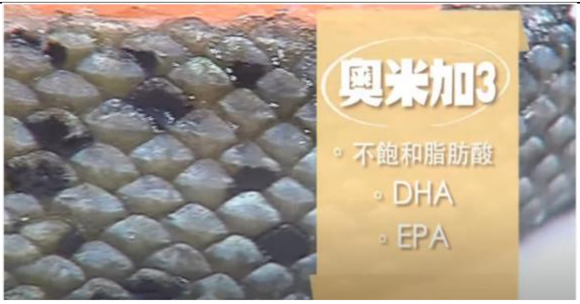

Example of healthy ingredients

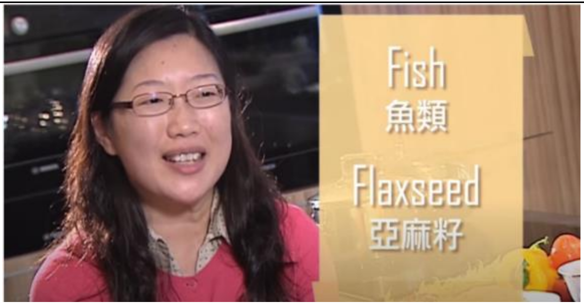

Example of healthy food

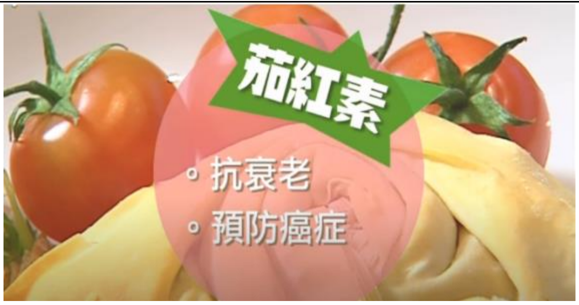

Example of healthy ingredients

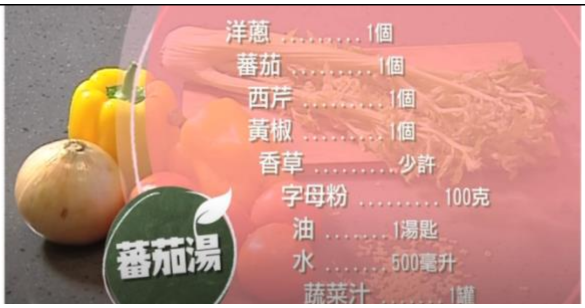

Menu for healthy food

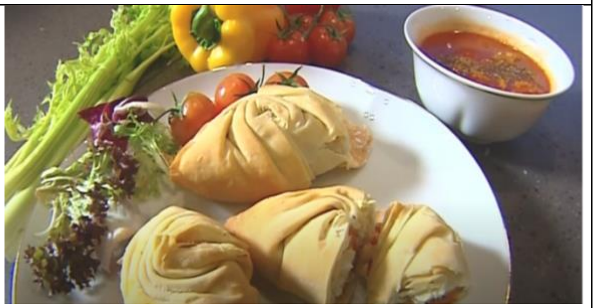

Example of healthy food

Supplementary file Figure 6 CONSORT flow diagram

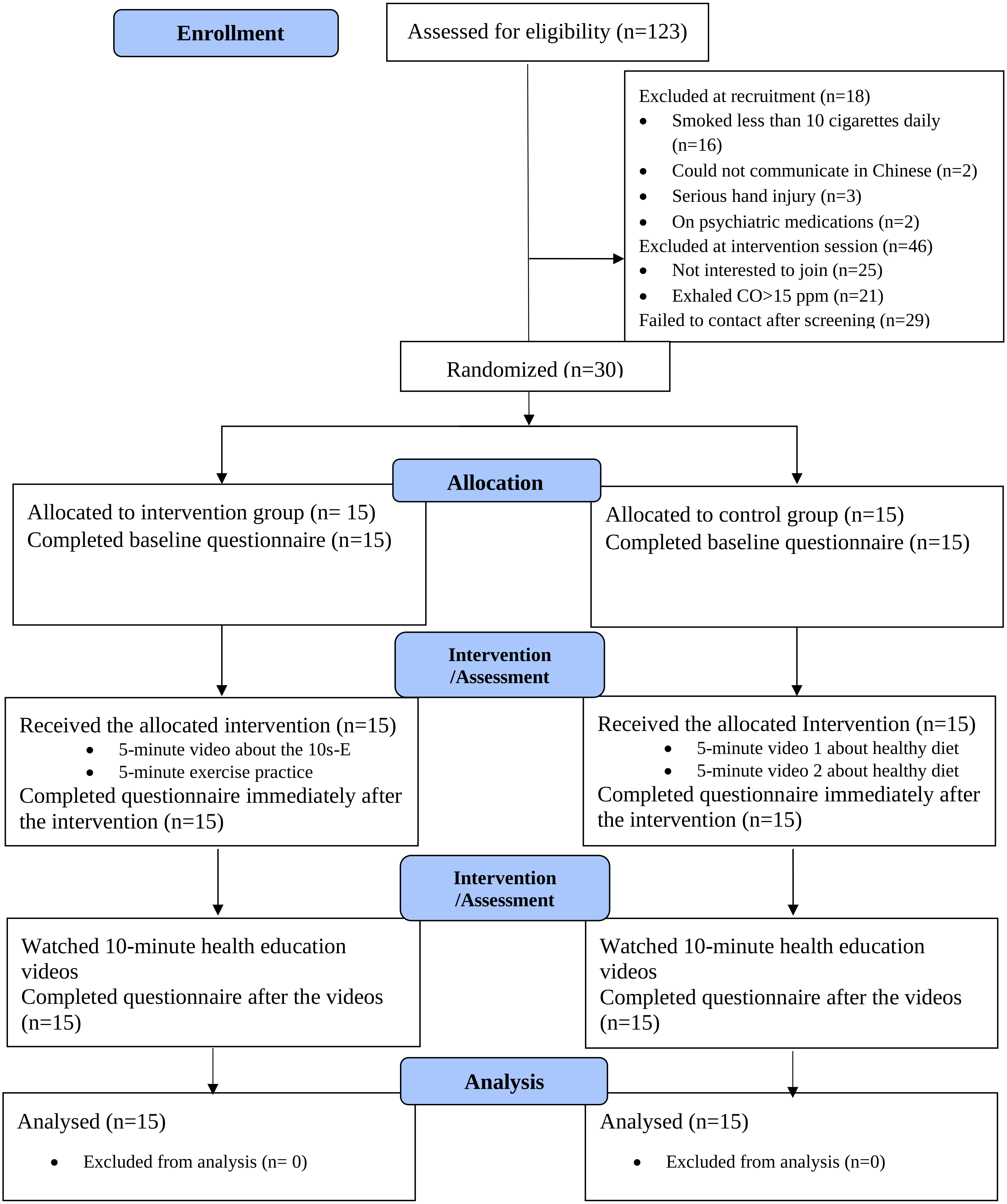

CONSORT 2010 checklist of information to include when reporting a randomised trial*

Section/Topic	Item No	Checklist item	Reported on page No
**Title and abstract**			
	1a	Identification as a randomised trial in the title	1
	1b	Structured summary of trial design, methods, results, and conclusions (for specific guidance see CONSORT for abstracts)	1
**Introduction**			
Background and objectives	2a	Scientific background and explanation of rationale	2-3
	2b	Specific objectives or hypotheses	3
**Methods**			
Trial design	3a	Description of trial design (such as parallel, factorial) including allocation ratio	4
	3b	Important changes to methods after trial commencement (such as eligibility criteria), with reasons	Not applicable
Participants	4a	Eligibility criteria for participants	4
	4b	Settings and locations where the data were collected	4-5
Interventions	5	The interventions for each group with sufficient details to allow replication, including how and when they were actually administered	6
Outcomes	6a	Completely defined pre-specified primary and secondary outcome measures, including how and when they were assessed	7-8
	6b	Any changes to trial outcomes after the trial commenced, with reasons	Not applicable
Sample size	7a	How sample size was determined	6
	7b	When applicable, explanation of any interim analyses and stopping guidelines	Not applicable
Randomisation:			5
Sequence generation	8a	Method used to generate the random allocation sequence	5
	8b	Type of randomisation; details of any restriction (such as blocking and block size)	5
Allocation concealment mechanism	9	Mechanism used to implement the random allocation sequence (such as sequentially numbered containers), describing any steps taken to conceal the sequence until interventions were assigned	5
Implementation	10	Who generated the random allocation sequence, who enrolled participants, and who assigned participants to interventions	5
Blinding	11a	If done, who was blinded after assignment to interventions (for example, participants, care providers, those	5

		assessing outcomes) and how	
	11b	If relevant, description of the similarity of interventions	Not applicable
Statistical methods	12a	Statistical methods used to compare groups for primary and secondary outcomes	8-9
	12b	Methods for additional analyses, such as subgroup analyses and adjusted analyses	8-9
**Results**			
Participant flow (a diagram is strongly recommended)	13a	For each group, the numbers of participants who were randomly assigned, received intended treatment, and were analysed for the primary outcome	Appendix Figure 6
	13b	For each group, losses and exclusions after randomisation, together with reasons	9
Recruitment	14a	Dates defining the periods of recruitment and follow-up	9
	14b	Why the trial ended or was stopped	Not applicable
Baseline data	15	A table showing baseline demographic and clinical characteristics for each group	Table 1
Numbers analysed	16	For each group, number of participants (denominator) included in each analysis and whether the analysis was by original assigned groups	Appendix Figure 6
Outcomes and estimation	17a	For each primary and secondary outcome, results for each group, and the estimated effect size and its precision (such as 95% confidence interval)	9-10
	17b	For binary outcomes, presentation of both absolute and relative effect sizes is recommended	Not applicable
Ancillary analyses	18	Results of any other analyses performed, including subgroup analyses and adjusted analyses, distinguishing pre-specified from exploratory	9-10
Harms	19	All important harms or unintended effects in each group (for specific guidance see CONSORT for harms)	Not applicable
**Discussion**			
Limitations	20	Trial limitations, addressing sources of potential bias, imprecision, and, if relevant, multiplicity of analyses	12
Generalisability	21	Generalisability (external validity, applicability) of the trial findings	11-12
Interpretation	22	Interpretation consistent with results, balancing benefits and harms, and considering other relevant evidence	10-11
**Other information**			
Registration	23	Registration number and name of trial registry	NCT04059497 in https://clinicaltrials.gov/
Protocol	24	Where the full trial protocol can be accessed, if available	Available upon request
Funding	25	Sources of funding and other support (such as supply of drugs), role of funders	13

Citation: Schulz KF, Altman DG, Moher D, for the CONSORT Group. CONSORT 2010 Statement: updated guidelines for reporting parallel group randomised trials. BMC Medicine. 2010;8:18.
© 2010 Schulz et al. This is an Open Access article distributed under the terms of the Creative Commons Attribution License (http://creativecommons.org/licenses/by/2.0), which permits unrestricted use, distribution, and reproduction in any medium, provided the original work is properly cited.

*We strongly recommend reading this statement in conjunction with the CONSORT 2010 Explanation and Elaboration for important clarifications on all the items. If relevant, we also recommend reading CONSORT extensions for cluster randomised trials, non-inferiority and equivalence trials, non-pharmacological treatments, herbal interventions, and pragmatic trials. Additional extensions are forthcoming: for those and for up-to-date references relevant to this checklist, see www.consort-statement.org.

© 2024 Zhang M.J. et al.
